# Solitary cutaneous mastocytoma of the scalp in adolescence: A rare presentation

**DOI:** 10.1016/j.jdcr.2026.04.009

**Published:** 2026-04-16

**Authors:** Yara Majed Alzahrani, Nouf Abdullatif AlDalaqan, Abdulelah Abdulhadi Aldossari, Lamia Alakrash

**Affiliations:** a General Physician, College of Medicine, Almaarefah University, Riyadh, Saudi Arabia; b Medical Doctor, College of Medicine, King Saud University, Riyadh, Saudi Arabia; c Resident Physician, Dermatology Department, King Fahad Specialist Hospital, Buraydah, Saudi Arabia; d Consultant Dermatologist, Department of Dermatology, King Fahad Medical City, Riyadh, Saudi Arabia

**Keywords:** CD117 (KIT), dermoscopy, mastocytosis darier sign, pediatric dermatology, scalp, solitary cutaneous mastocytoma

## Introduction

Mastocytosis is a heterogenous disease marked by abnormal growth of mast cells accumulated in skin or other organs.^1^ WHO classifies mastocytosis into 2 forms: systemic and cutaneous, systemic mastocytosis typically affects adults with extracutaneous involvement, while cutaneous mastocytosis mainly affects children and includes several clinical variants (eg, maculopapular cutaneous mastocytosis/urticaria pigmentosa, diffuse cutaneous mastocytosis, and solitary mastocytoma).^2^ Mastocytoma presents as a diagnostic challenge due to the variety and non-specific symptoms such as pruritus, flushing, gastrointestinal complaints, hypotension, or localized skin lesions.^3^ Typically characterized by a reddish-brown macules or papules that often have positive Darier’s.^4^ Cutaneous mastocytoma commonly involve the trunk and extremities however, mastocytoma on the scalp in adolescents and young adults appears to be uncommon and rarely described.^1^^,^^3^^,^^4^ This report aims to highlight the rare appearance of solitary cutaneous mastocytoma in adolescence located on scalp and the importance of examination with histopathological characteristics in dermatological practice to distinguish it from other skin disorders.

## Case presentation

A 10-year-old girl presented with long-standing scalp pruritus and intermittent facial flushing precipitated by heat, hair brushing, mechanical friction and emotional tension. Review of systems was negative for syncope, respiratory distress, or gastrointestinal symptoms. The lesion had been present since infancy and was previously misdiagnosed clinically as an infantile hemangioma. The lesion size remained stable over time without bleeding or ulceration.

Physical examination revealed a solitary, firm, non-tender, tan, dome-shaped plaque (approximately 2 × 1 cm) on the scalp vertex.

**Fig 1:**
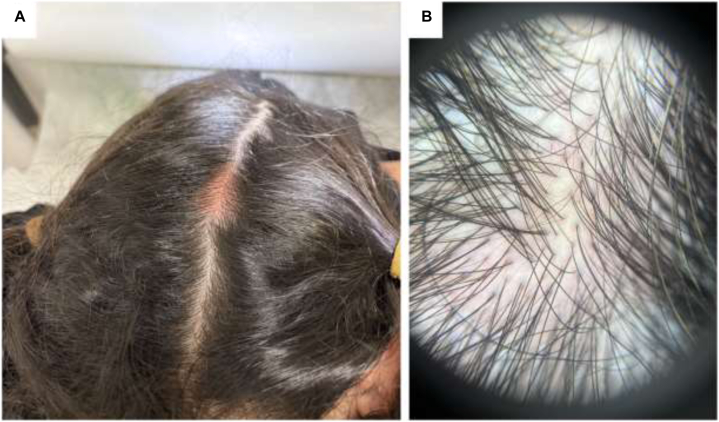
**A,** Clinical and dermoscopic presentation. **A,** Clinical photograph showing a solitary, well-circumscribed, erythematous to tan-colored edematous plaque on the vertex scalp. **B,** Dermoscopy reveals a homogenous, structureless pattern with a subtle *yellowish-pink* background.

On dermoscopy, the lesion displayed a structureless yellow-pink background with preserved follicular ostia and hair density, lacking the vascular features typical of hemangiomas (Fig 1, *A*). Stroking the lesion elicited an urticarial reaction (positive Darier sign). No other cutaneous lesions were observed.

**Fig 3:**
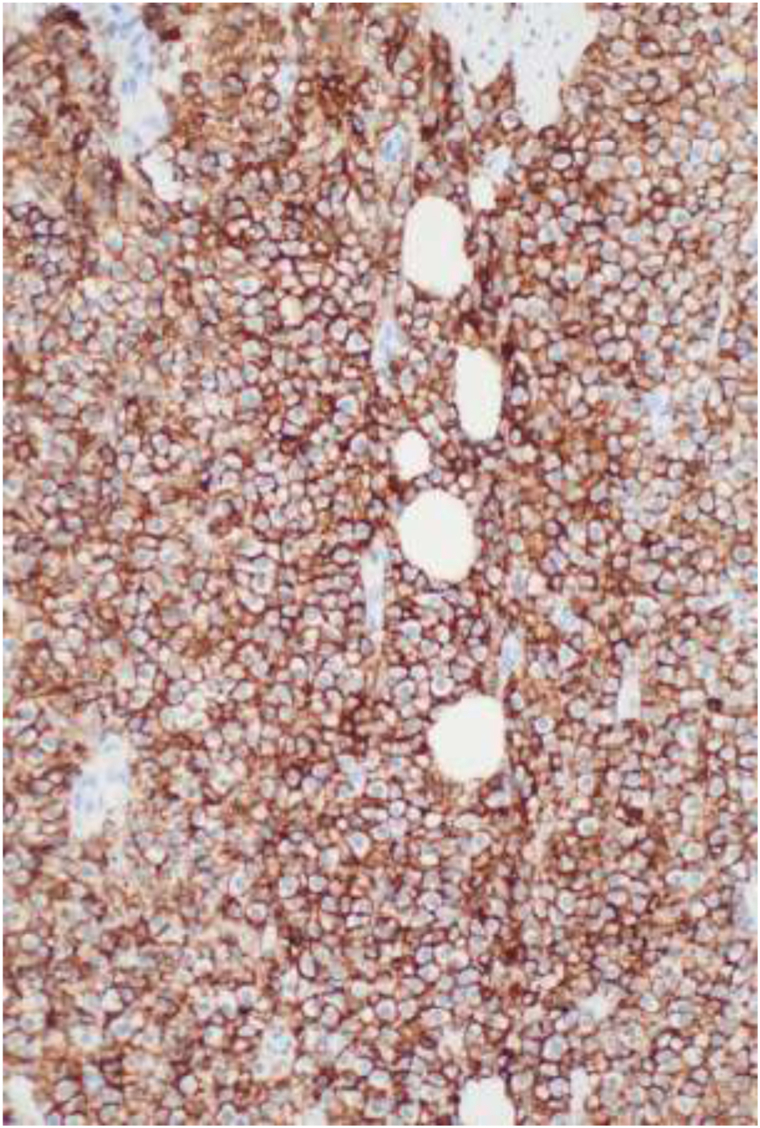
Diffuse, dense dermal infiltrate of mast cells showing strong, uniform cytoplasmic and membranous positivity for CD117/KIT under imunnohistochemistry staining.

**Fig 2:**
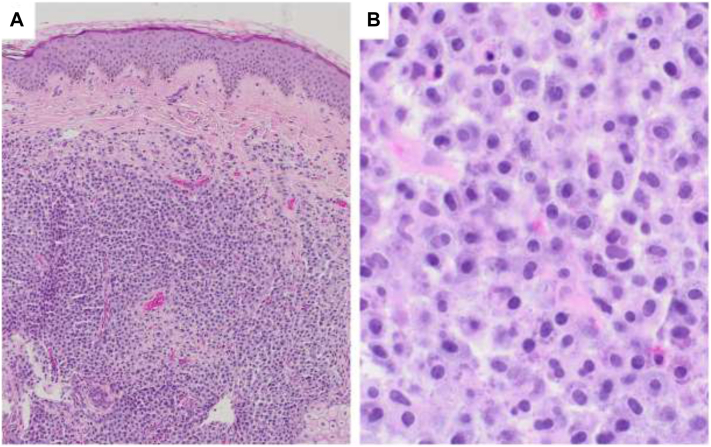
**A,** Low-power view demonstrating a normal epidermis separated from the underlying dermal infiltrate by a distinct Grenz zone. The mid- and reticular dermis show a dense, diffuse infiltration predominantly composed of mast cells. **B,** Higher magnification of mast cells with cytoplasmic granules and classical fried-egg appearance.

Histopathologic examination demonstrated a dense dermal mast cell infiltrate (Fig 2) with diffuse CD117 positivity (Fig 3), confirming a solitary cutaneous mastocytoma. Subsequent serum tryptase was normal (7.3 ng/mL), helping to exclude systemic mastocytosis.

The patient reported symptomatic relief with daily loratadine and trigger avoidance.

## Discussion

Solitary cutaneous mastocytoma (SCM) is the most common type of childhood mastocytosis and occurs most often in infancy, often regressing before adolescence.^5^^,^^6^

Scalp involvement is rare, with a few pediatric scalp cases mentioned in the literature, which might explain delayed detection or misdiagnosis. Because a few pediatric scalp cases have been reported, no consistence sex predilection or other specific epidemiological pattern can currently be concluded for this uncommon presentation.^6^

This case is notable for its scalp location and persistent manifestation into adolescence. The patient’s episodic facial flushing and chronic pruritus elicited by heat, friction, grooming, and stress are consistent with mast cell mediator release and similar provocation following manipulation or procedures has been described. With a broad differential diagnosis for only a single scalp plaque/nodule in childhood, histopathologic confirmation is especially beneficial in atypical sites or prolonged courses.

In our case, biopsy revealed a dense dermal mast cell infiltrate (Fig 2, *A* and *B*) with strong diffuse CD117 (KIT) positivity (Fig 3), indicating SCM.^5^^,^^6^

In children evaluation should be symptom-directed. Baseline testing (eg, CBC, liver function tests, serum tryptase) is reasonable, and ongoing workup should be limited to systemic symptoms, organomegaly, or persistently elevated tryptase.^4^^,^^6^

Additional reported cases of solitary mastocytoma, including unusual presentations in adults and pediatric patients with recurrent blistering, urticaria, or angioedema, further demonstrate the variable clinical spectrum of this condition.^7^, ^8^, ^9^

Management is largely preventive and symptomatic: trigger avoidance and oral H1 antihistamines to control pruritus and flushing; excision in general reserved for diagnostic uncertainty, persistent symptoms, or repeated trauma.^6^^,^^10^

This case fills a narrow pediatric literature regarding scalp SCM and highlights the clinical manifestation of persistent, symptomatic lesions beyond early childhood. Clinicians should look into SCM in cases of solitary scalp lesions that involve episodic urtication/flushing, confirm atypical cases with mast cell–directed histology/IHC, and provide focused counseling on friction-related triggers.^4^, ^5^, ^6^

## Conclusion

Although rare, scalp mastocytoma should be considered in the differential diagnosis of solitary cutaneous lesions in pediatric patients. Early recognition based on clinical features and confirmatory histopathology ensures appropriate management and help avoid unnecessary investigations or interventions.

### Declaration of generative AI and AI-assisted technologies in the writing process

During the preparation of this manuscript, the authors used (OpenAI) to assist with language editing and organization and Grammer accuracy The authors reviewed, verified, and edited the content as necessary and take full responsibility for the accuracy and integrity of the final manuscript.

## Conflicts of interest

None disclosed.
